# Navigating therapeutic strategies: HPV classification in head and neck cancer

**DOI:** 10.1038/s41416-024-02655-1

**Published:** 2024-04-20

**Authors:** Hossein Tabatabaeian, Yuchen Bai, Ruihong Huang, Akhilanand Chaurasia, Charbel Darido

**Affiliations:** 1 Peter MacCallum Cancer Centre, 305 Grattan St, Melbourne, VIC Australia; 2 Department of Oral Medicine and Radiology, Faculty of Dental Sciences King George’s Medical University, Lucknow, Uttar Pradesh India; 3 The Sir Peter MacCallum Department of Oncology, The University of Melbourne, Parkville, VIC Australia

**Keywords:** Oral cancer, Cell signalling, Head and neck cancer

## Abstract

The World Health Organisation recognised human papillomavirus (HPV) as the cause of multiple cancers, including head and neck cancers. HPV is a double-stranded DNA virus, and its viral gene expression can be controlled after infection by cellular and viral promoters. In cancer cells, the HPV genome is detected as either integrated into the host genome, episomal (extrachromosomal), or a mixture of integrated and episomal. Viral integration requires the breakage of both viral and host DNA, and the integration rate correlates with the level of DNA damage. Interestingly, patients with HPV-positive head and neck cancers generally have a good prognosis except for a group of patients with fully integrated HPV who show worst clinical outcomes. Those patients present with lowered expression of viral genes and limited infiltration of cytotoxic T cells. An impediment to effective therapy applications in the clinic is the sole testing for HPV positivity without considering the HPV integration status. This review will discuss HPV integration as a potential determinant of response to therapies in head and neck cancers and highlight to the field a novel therapeutic avenue that would reduce the cancer burden and improve patient survival.

## Background

Head and neck squamous cell carcinomas (HNSCC) is the main type of malignancy of the head and neck region and the seventh most common cancer worldwide accounting for nearly 1 million new cases, and 470,000 deaths in 2020 [1]. The incidence of HNSCC continues to rise and is anticipated to reach an annual incidence rate of 1.37 million new cases by 2040 [2, 3].

HNSCC originates from the mucosal epithelium of the lips, oral cavity, salivary glands, larynx and pharynx. The major external risk factors associated with the incidence of HNSCC are exposure to excessive alcohol consumption, tobacco-related cancer-causing agents, or both. Along with these risk factors, intrinsic genetic, transcriptional and post-translational changes such as the expression and/or function of Fanconi Anaemia Complementation Group (FANC) [4], Grainyhead-like 3 (GRHL3) [5], Filaggrin (FLG) [6], and cellular localisation of Y-box binding protein 1 (YBX1) [7] impact HNSCC development. On the other hand, oropharyngeal squamous cell carcinoma (SCC) tumours are associated with prior infection with oncogenic strains of human papillomavirus (HPV), whereas >70% of oropharyngeal cancers are HPV-positive. Studies reveal a global trend of rising HPV-related cancers and declining HPV-unrelated subsites. Over the next two decades, the majority of HNSCC cases are expected to be HPV-positive, with some countries like the UK experiencing a higher incidence of oropharyngeal cancer than oral cavity cancer [8, 9].

In this review, we summarise the role of HPV infection in head and neck cancer development, with a focus on chromosomal abnormalities and the viral integration process. We also highlight how the integration status affects the patients’ therapeutic outcomes.

## HPV: a virus causing cancer in humans

HPV is the most prevalent sexually transmitted infection worldwide that has significant social implications. Both sexually active women and men are susceptible to HPV infection [10], although not all individuals will develop associated health issues. HPV infection remains widespread worldwide as one of the leading causes of cancer [11], especially among women, making it a critical concern for public health.

HPV has different strains, each identified with a number. The strains are classified into two categories: low-risk HPVs responsible for anogenital and cutaneous warts, and high-risk HPVs responsible for oropharyngeal and anogenital cancers [12–16]. HPV16 is the highest-risk strain accounting for more than 90% of HPV-associated HNSCC, while other HPV strains such as HPV-18, 31, 33 and 52 have been detected in a small proportion of patients [17–20].

The currently approved HPV vaccines, including bivalent, tetravalent, and 9-valent vaccines, have demonstrated effectiveness in reducing both HPV infection and the incidence of HPV-related diseases across various geographical regions worldwide. The vaccines target and stimulate immunity against both low- and high-risk HPVs, which are responsible for most genital and cutaneous warts (70%) as well as HPV-positive cancers (90%). Despite the proven efficacy of HPV vaccines, the burden of HPV-associated cancers and diseases remains substantial [21, 22]. Therefore, epidemiological surveillance of HPV infection and related diseases is crucial in this space. This surveillance would play a vital role in monitoring and evaluating the effectiveness of available antiviral prophylactic vaccines and assessing their acceptance globally.

## Molecular mechanisms involved in the cycle of HPV replication

**Fig. 1:**
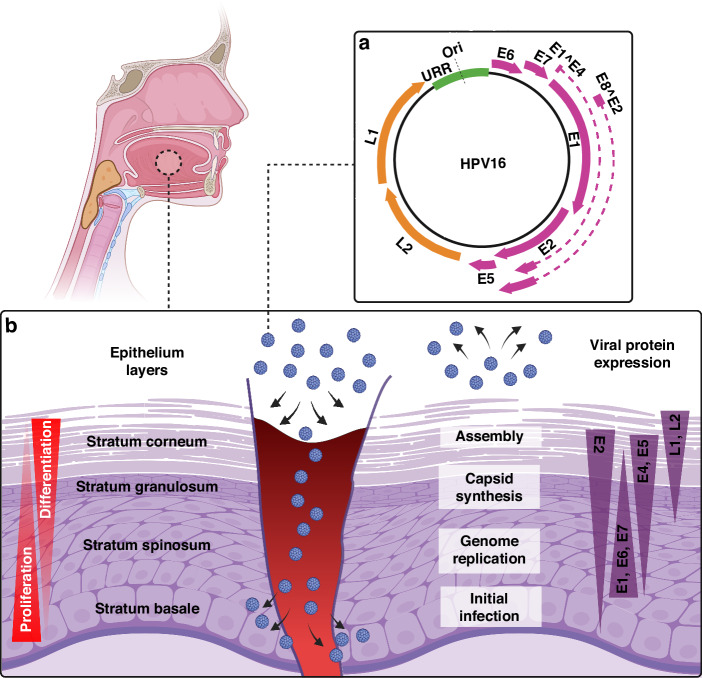
HPV16 genome structure and life cycle. **a** Structural late L1 and L2 genes, and executive early E1–E8 genes are expressed from a nearly 8 Kbp HPV16 genome. **b** The HPV infection starts in the basal epithelial cells upon injury/trauma/permeability and ends with the assembly and virion release at the very top terminally differentiated epithelial cells. Early genes of E1, E2, E6, E7, E4, E5 and late genes of L1 and L2 are expressed in basal to superficial layers during the infection initiation, progression and termination. Ori origin of replication, URR upstream regulatory region.

HPV is a small (50–60 nm in diameter), non-enveloped, double-stranded DNA virus belonging to the Papillomaviridae family. Unidirectional transcription of one strand of the 7–8 kb circular genomic DNA encodes for eight functional early (E1–E8), two structural late (L1 and L2) proteins [23], and a long non-coding control region also known as upstream regulatory region (URR) (Fig. 1a). Brief characteristics of expressed HPV genes are reviewed by Van Doorslaer et al. [24] and summarised in Table 1.

**Table 1:** Characteristics of functional early and late structural HPV genes.

Gene	Role	Reference
E1	Ori-binding DNA helicase required for replication and amplification of the virus.	[101]
E2	Master regulator of the viral life cycle Initiating the replication process and partitioning of the viral genome. Functioning as a transcriptional activator or repressor. Participating in virus genome segregation over host cell mitosis process.	[23, 102, 103]
E1^E4	Being expressed at the late stage of viral infection to facilitate the virus leaving the host cell by disrupting the host cytokeratin network.	[104–107]
E5	Oncogenic small hydrophobic transmembrane proteins playing a role in the productive virus life cycle.	[44]
E6	Oncogenic protein inhibiting p53 protein via a direct physical interaction. Preparing cellular environment to allow for replication and immortalisation.	[28]
E7	Oncogenic protein inhibiting Rb protein via a direct physical interaction. Preparing cellular environment to allow for replication and immortalisation.	[29]
E8^E2	Inhibiting viral replication and gene expression.	[108]
L1	Being expressed upon cellular differentiation. Main structural component of the viral capsid.	[109]
L2	Expressed upon cellular differentiation. Minor capsid protein playing an active role in the assembly of HPV virus throughout the infectious process.	[110]

*HPV* human papillomavirus, *Ori* origin of replication, ORF open-reading frame.

The infection mechanism of HPV, despite the genetic variations among different strains and their diverse infection profiles, follows a similar pattern. HPV specifically targets basal epithelial cells, homed in the undifferentiated deeper layers of squamous epithelia and/or mucous membranes and possess a high mitotic capacity. To reach these target cells, the virus presence on the outer apical surface of these tissues relies on microlesions that occur during traumatic events. Upon reaching the basal epithelial cells, the virus is internalised by endocytosis. It is then transported within small vesicles through the endoplasmic reticulum (ER) and the Golgi apparatus. During this journey, a series of interactions and structural changes in the vesicles occur, leading to the removal of the viral capsid and the release of the viral genome near the nuclear membrane [25].

The viral genome enters the nucleus via nuclear pores and remains in the form of an episome (i.e., extrachromosomal) while the process of viral replication occurs via the expression and function of E1, E2, E5, E6 and E7 genes [26, 27]. Among the genes transcribed early in the viral life cycle, E2, E5, E6 and E7 play crucial roles in hijacking the cellular environment that is conducive to viral DNA replication [28, 29]. The chronology and level of HPV gene expression progress from the basal to suprabasal epithelial cells with an ultimate virus assembly in the terminally differentiated cells as presented in Fig. 1b. Of note, E6 and E7 interfere with cellular function by inhibiting two important tumour suppressor genes, i.e., p53 and Rb, to promote the proliferation of infected cells. E6 causes p53 protein degradation via mediating its interaction with E6-associated protein (E6-AP) E3-ubiquitin ligase [30]. Similarly, Rb1 undergoes sequestration and proteolysis due to E7 protein [31].

The transcriptional regulation of all functional and structural HPV genes is modulated via the upstream regulatory region (URR). The URR induces the transcription of downstream genes to start the viral genome replication upon binding of viral E1 and E2 proteins. This process is also activated by host cell transcription factors, such as nuclear factor 1 (NFI), activator protein 1 (AP-1), organic cationic transporter 1 (Oct-1), Translation elongation factor 1 (TEF-1) (also known as TF1), and specificity protein 1 (SP1) [32]. In addition to the transcriptional role of URR, the origin of replication (Ori) site lies within this region. Ori is pivotal for papillomavirus replication and is highly homologous to the mammalian autonomous replicating conserved sequences (ACS) [33]. Each papillomavirus genome contains only one Ori required for viral DNA replication [34].

To summarise, insights into the molecular characteristics and evolution of HPV infection is fundamental for understanding the distribution of HPV and its impact on HPV-related diseases. This knowledge can inform the development of new therapeutic strategies and next-generation antiviral vaccines that address the limitations of the current prophylactic regimens, such as high costs, limited spectrum of antiviral protection, and immunisation management.

## Molecular differences between HPV-negative and positive HNSCC

HPV-positive HNSCC displays notable variations compared to HPV-negative HNSCC regarding immune characteristics, gene expression and importantly, mutational patterns, highlighting the distinctive entity of HPV-positive HNSCC.

### Immune system and microenvironment

The composition and abundance of immune cells in tumours differ significantly between HPV-positive and HPV-negative cases [35, 36]. HPV-positive tumours generally exhibit higher levels of tumour-infiltrating lymphocytes (TILs) compared to HPV-negative tumours. Notably, patients with HPV-positive tumours containing a high number of TILs (CD8^+^ cytotoxic T cells) tend to have favourable outcomes, while those with HPV-positive tumours and low TIL levels experience similar poor survival rates to patients with HPV-negative HNSCC [37].

HNSCC tumours manifest various mechanisms to evade immune surveillance. The tumour microenvironment (TME) in HNSCC is enriched with immunosuppressive growth factors and cytokines that promote the recruitment or activity of myeloid-derived suppressor cells (MDSCs), regulatory T cells (Treg cells), and M2 macrophages. Simultaneously, these factors hinder the anti-tumour effects of effector T cells (T_eff_ cells) and natural killer (NK) cells. Notably, cytokines such as IL-6, IL-10, VEGF and TGF-β play a crucial role in this immunosuppressive environment [38]. Elements within the HNSCC TME, including IL-10 and TGFβ, contribute to the polarisation of macrophages into an immunosuppressive M2 phenotype [39]. Genetic and epigenetic alterations further reduce the levels of human leucocyte antigen (HLA) on tumour cells and impair antigen processing [40, 41]. Consequently, tumour cells evade recognition and elimination by the immune system. Moreover, HNSCC tumours, particularly in advanced stages, exhibit increased expression of programmed death-ligand 1 (PDL1), which suppresses the cytolytic activity of T cells [42, 43]. Likewise, MDSCs and Treg cells recruited to the HNSCC TME express PDL1 and cytotoxic T lymphocyte antigen 4 (CTLA4), respectively, both of which are immunosuppressive molecules. In the context of HPV-positive HNSCC, limited knowledge on the patient immunological status compared to HPV-negative HNSCC is available. Nevertheless, it is known that in HPV-positive HNSCC, the viral proteins E5, E6 and E7 promote immune evasion by altering the expression profile of tumour cell proteins [44, 45]. In addition, the frequent loss of TRAF3, a gene involved in antiviral immunity in HPV-positive HNSCC likely contributes to immune evasion [46, 47]. More comprehensive studies are needed in this space to understand the immune cell regulation associated with HPV infection which would shed new light for the identification of treatment strategies against HPV-positive HNSCC.

### Genetic alterations

Cumulative evidence has reported a large difference between the mutational signature of HPV-negative and positive tumours, due to E6 and E7 inhibitory functions of key tumour suppressor genes *TP53* and *RB1*. The key distinctions between HPV-negative and HPV-positive HNSCC are as follows: HPV-negative HNSCCs exhibit a high prevalence of mutations in *CDKN2A*, *TP53*, *FAT1*, *NOTCH1*, *CASP8*, *HRAS* and loss of chromosome region *3p*. On the other hand, HPV-positive patients demonstrate high mutation rates in *PIK3CA*, *ZNF750*, *EP300*, *CYLD*, *TRAF3*, *FGFR3*, *PTEN*, *B2M* and *RB1*, along with gain of chromosome regions *3q* and *19q*, and loss of chromosome regions *11q*, *16q* and *18q*. Although the top mutated genes in HPV-negative tumours are *CDKN2A* and *TP53*, loss of *TRAF3* and amplification of *E2F1* are the most frequently mutated genes in HPV-positive tumours [48]. Moreover, the occurrence of WGD and WGT events is significantly higher in HPV-negative cases compared to HPV-positive HNSCC. These events tend to happen earlier in the development of HPV-positive HNSCC [49]. This stark contrast suggests that the initiation and progression of HNSCC are mechanistically different depending on the presence or absence of HPV infection.

**Fig. 2:**
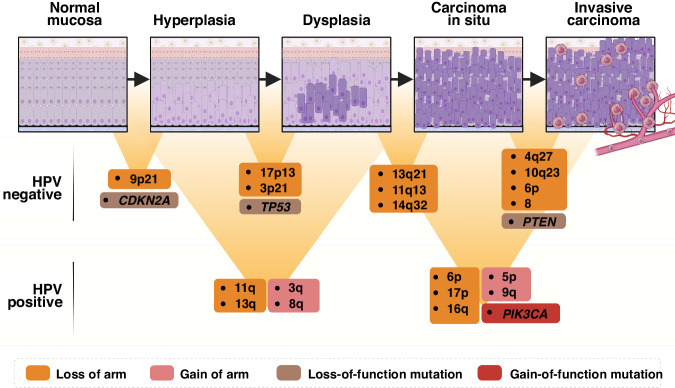
Chronological genetic alterations in HPV-negative and HPV-positive HNSCC. Major differences in the frequency of genetic lesions in HPV-negative and positive HNSCC are shown. HPV human papillomavirus, CDKN2A cyclin-dependent kinase inhibitor 2A, TP53 tumour protein 53, PTEN phosphatase and tensin homologue, PIK3CA phosphatidylinositol-4,5-bisphosphate 3-kinase catalytic subunit alpha.

In contrast to the HPV-positive tumours, the chronological order of genetic events in HPV-negative HNSCC has been well-documented owing to available premalignant and malignant lesions throughout the steps of cancer progression from hyperplasia to invasive carcinoma [50, 51] (Fig. 2). Loss of *9p21* locus that harbours *CDKN2A* and *ARF* tumour suppressor genes occurs in the very early stages of cancer initiation from the normal mucosa to hyperplasia. These genes encode for the CDK4 and CDK6 inhibitor p16INK4A and a stabiliser of p53 protein p14. The transition to the dysplastic stage is coincident with the loss of *TP53*-containing locus *17p13* and *3p21*. Loss of *13q21*, *11q13* and *14q32* loci further facilitate the transition to carcinoma in situ, and frequent losses in chromosomes *4q27, 8, 6p* and *10q23* are prevalent in invasive primary tumours [20, 48].

Despite well-defined genetic changes occurring during the progression of HPV-negative HNSCC, chronological genetic alterations in HPV-positive cancers are still less understood [52]. This knowledge gap can be attributed to the challenges associated with identifying HPV-positive premalignant lesions [52, 53]. To address this issue, Leshchiner et al. employed the PhylogicNDT LeagueModel bioinformatic tool to investigate potential genetic abnormalities in 101 HPV-positive HNSCCs using whole exome sequencing (WES) data. By comparing the bioinformatic findings to alterations observed in HPV-negative cases, the authors validated the algorithm’s effectiveness in predicting pre-malignancy mutations in 421 HPV-negative HNSCCs. Of note, PhylogicNDT identified loss of *9p* (*CDKN2A* inactivation) and *3p* and mutations in *TP53* as early events for HPV-negative tumour development. In addition to the sequential events detected by PhylogicNDT, whole genome duplication and triplication (WGD and WGT) were reported to occur late during the progression to HNSCC [49].

This approach was then utilised to investigate the genetic events occurring in HPV-positive HNSCC, aiming to identify the genetic alterations in early and late-stage tumour development. In HPV-positive HNSCC, specific changes such as amplification of chromosome regions *3q* and *8q*, coupled with the loss of chromosome regions *11q* and *13q*, are frequently observed in the early stages. In addition, the loss of chromosome regions *6p*, *17p* and *16q*, as well as the amplification of chromosome regions *5p* and *9q*, along with mutations in the *PIK3CA* gene, are highly prevalent in the advanced stages of HPV-positive HNSCC progression (Fig. 2). However, this approach has not accurately predicted the specific genetic alterations that occur during the transition from hyperplasia to dysplasia, dysplasia to carcinoma in situ and invasive HNSCC.

In a more recent comprehensive study, Cheng et al. performed integrated analyses of genomic and transcriptomic profiles of 15 human HPV-negative and 11 HPV-positive HNSCC cell lines [54]. They compared their results with publicly available RNA-seq and genomics data obtained from 279 HNSCC tumours from The Cancer Genome Atlas (TCGA) [48]. In this study, the regions subjected to recurrent alterations were meaningful as they are confirmed in multiple samples, contrary to the individual patient-specific alterations discussed before. Chromosomal gains in *3q*, *5p, 7p, 8q* and *11q* and losses in *3p, 5q, 8p, 9p, 10p, 18q* and *21q* were consistently detected in genomics analyses of both HNSCC TCGA and HNSCC cell lines [54].

Regardless of HPV infection, gain of *3q26–q28* harbouring *PIK3CA*, sex-determining region Y-box 2 (*SOX2*), *TP63*, and Telomerase RNA Component (*TERC*) and gain of *11q* are the dominant genomic alterations. Consistent with TCGA data, a gain of chromosome *11q* that harbours Fas-Associated protein with Death Domain (*FADD*), Cyclin D1 (*CCND1*), Cortactin (*CTTN*), Fibroblast Growth Factor (*FGF3*/*4*/*19*), Protein Tyrosine Phosphatase, Receptor Type, F Interacting Protein Alpha 1 (*PPFIA1*), Myeloma Overexpressed (*MYEOV*), and Anoctamin 1 (*ANO1*), and gain of *11q22* that contains Baculoviral IAP Repeat Containing (*BIRC2*/*3*), Yes-associated protein (*YAP1*), and Platelet-Derived Growth Factor D (*PDGFD*), and loss of *9p21* that carries *CDKN2A* were predominately amplified in HPV-negative lines. Copy number alterations combined with transcriptomic analyses further revealed that gains in *3q, 11q* and *5p* increase the expression levels of HNSCC-related oncogenes. Chromosome *3q26* loci play a pivotal role as its amplification and *TP53* mutation coincidence significantly correlate with the poor prognosis of HNSCC patients [54]. These findings collectively confirm that the HNSCC cell lines recapitulate genomic alterations of more aggressive HNSCC tumour subtypes, thus the cell lines might not be the ideal models to study cancer initiation.

## HPV integration is a dynamic process during the tumourigenic process

**Fig. 3:**
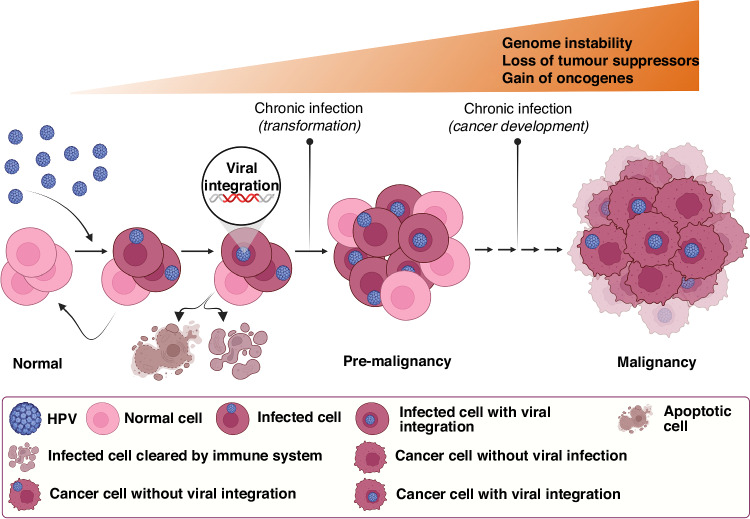
The carcinogenesis process is triggered by HPV integration into the host cell genome. Following an infection with HPV, the integration process could take place in infected cells that evade the immune system and are not cleared by apoptosis. During chronic infection, infected cells can become immortalised (transformed) and by accumulating various alterations in tumour suppressors and oncogenes eventually progress to cancer. The HPV-positive pre-cancer and cancer cells harbour integrated and non-integrated HPV. The HPV-positive cells with a higher rate of viral integration are enriched at the final stages of HNSCC and correlate with a poor patient prognosis. HPV human papillomavirus, HNSCC head and neck cancer squamous cell carcinoma.

HPV integration refers to the process by which the HPV DNA becomes physically integrated into the host cell’s genome. The identification of multiple integration sites within early gains in HPV-positive HNSCC suggests that the integration process is a precursor to the genetic alterations observed during tumorigenesis. Notably, the integration events can occur as early as 25 years before the onset of primary tumours and many years before the WGD/WGT occurs. These highlight that HPV integration is the main contributor to HPV-positive cancer initiation [49]. Notwithstanding, the integration traces may or may not remain throughout the cancer development. For instance, Parfenov et al. [55] detected HPV integration in 25 of the 35 studied cases, meaning that nearly 30% of HPV-positive tumours had only HPV episomes. In addition, the detection of the viral genome or its fragments, integrated into the genome of high-grade HNSCC [55, 56] and cervical cancers [57–60] suggest that the integration process occurs more frequently in the late stages of cancer progression. In line with this, the heightened integration levels of HPV16 into the host genome of 56 TCGA HPV-positive HNSCC correlates with advanced stages of cancer and poorer survival of HNSCC patients [61]. Thus, although the integration would start many years prior to tumour formation, HPV-positive cancer cells with viral integration status may be enhanced in advanced HNSCC (Fig. 3). This is in line with the analysis of integration sites in subclonal samples by PhylogicNDT revealing that integration is a dynamic process in HPV-positive HNSCC [49]. Furthermore, Puram et al. recently studied the HPV-positive oropharyngeal SCC samples at the single-cell resolution. Analysing the HPV transcripts in subclones of malignant epithelial single cells revealed that not all HPV-positive tumour cells express HPV genes. For instance, HPV was detected in 90%, 4% and 0% of cells in three subclones. Considering all the malignant epithelial cells from 11 samples showed reads of HPV in only 66% of HPV-positive samples. Besides differences in the frequency of HPV detection, some subclones differed in the relative detection of distinct HPV genes as well. Therefore, the overall frequency and relative expression patterns of HPV genes are subject to changes during tumour evolution [62].

## HPV integration is facilitated by DNA damage during the progression of HPV-positive HNSCC

During the process of viral DNA integration, both viral and host cell genomes undergo a molecular breakage. Akagi et al. discovered that breakpoints occurred throughout the viral genome, leading to the reduction of E2 and retainment of E6 and E7 in a mixture of HPV16-positive cervical and HNSCC tumours [63]. E2 plays a pivotal role in maintaining the life cycle of HPV by initiating DNA replication. It is also known that the E2 protein suppresses the expression and function of E6 and E7 oncoproteins [64–66], and induces apoptosis of infected cells [67–69]. Disruption of the E2 open-reading frame (ORF) is one of the main events caused by HPV integration, which eventually leads to enhanced expression of E6 and E7, thereby promoting cell growth [65, 70]. Along with this critical event, the integration process confers transforming capabilities to the host cells by stabilising the viral transcripts (produced from the integrated gene vs. episome) and also by deregulating the host genome/transcriptome depending on the integration sites [55, 71–73].

The rate of breakpoints in the host genome is very low (less than ten breakpoints in the genome), suggesting that a broader fragmentation in the host genome might be necessary for the full integration of the viral genome. Consistently, Parfenov et al. demonstrated that breakpoints of the host cell genome also tend to occur in regions of microhomology (1–10 bp) among the viral and host genome, and most frequently into genic or near-genic and miRNA regions [55]. Although the viral integration is linked to an increase in somatic DNA copy number of the integrated region, there is no significant association between the integration and mutational burden, indicating that HPV infection enhances the mutational burden in HNSCC regardless of integration. There is also no correlation between the occurrence of integration and the stage of cancer in the studied HPV-positive HNSCCs, which is in contrast with results showing an association between higher stages and poorer prognosis of HNSCCs and heightened levels of integration [55, 56, 61]. Consistent with other work, higher levels of E2 and E5 expression, and lower levels of E6 and E7 expression are observed in tumours without integration compared with integration-positive tumours. The overall results of these studies need further assessments as the outcomes have been generated from a small set of samples, i.e., *n* = 3 HPV-positive HNSCC + 2 HPV-positive cervical lines + 2 HPV-positive primary HNSCC tumours in Akagi et al.’s; and *n* = 35 HPV-positive HNSCCs in Parfenov et al.’s studies.

## HPV genome integration is associated with genetic alterations in HNSCC

The overall mutational burden in HPV-negative and -positive HNSCCs was reported to be in the same range, while many other groups have reported a higher load in HPV-negative cases [74–76]. Of note, in HPV-positive HNSCCs, the elevated frequency of mutations, deletions, amplifications, and structural translocations at sites of HPV integration was reported [77]. While the integration process seems to be random, Akagi et al. found HPV integrants cluster at specific regions in different cell lines. For example, the integrants cluster at chromosome *3p11* in UM-SCC-47, chromosome *Xq21* in UD-SCC-2 cells, and chromosomes *3p12, 6p21* and *9q22* in SCC090 cells. This highlights that cell-specific vulnerability might depend on the genetic and epigenetic backgrounds. These alterations eventually lead to the amplification and expression of integrated E6 and E7 viral genes and thereby the genomic instability of host cells. The key cancer-related genes affected by HPV integrants are Diaphanous-related formin 2 (*DIAPH2*) gene rearrangements and Tumour protein 63 (*TP63*) gene promoter aberration and Proviral integration site for Moloney murine leukaemia virus 1 (*PIM1*) and Forkhead box E1 (*FOXE1*) gene amplification [63].

Parfenov et al. reported the integrants to propose critical mechanisms by which the integration process could initiate tumorigenesis. A virus-host genome rearrangement resulting in the disruption of viral E1, E4 and E5 and the human RecA homologue 51B (*RAD51B*) gene could be one potential path to induce cancer development. Insertion of the HPV16 genome to the coding regions of E26 transformation-specific 2 (*ETS2*), and programmed death-ligand 1 (*PDL1*) was proposed as the second potential mechanism. Moreover, HPV integrants upstream of nuclear receptor subfamily 4, group A, member 2 (*NR4A2*) gene with subsequent effects on the amplification and overexpression of this oncogene were reported as key genetic changes in one of the HPV-positive HNSCC patients. Interestingly, the expression levels of E6 and E7 were very low, suggesting that such integration-mediated genetic change can override the roles of viral oncogenes. Another interesting mode of action was related to the insertion of HPV16 in nongenic regions which results in intrachromosomal rearrangements between chromosomes 3 and 13. Such remodelling affects TP63 regulated 1 (*TPRG1*) and *TP63* genes on chromosome 3 and the Kruppel-like factor 5 (*KLF5*) on chromosome 13. Integration-mediated overexpression of these genes could therefore drive tumourigenesis [55]. The HPV16 integration-mediated changes on the *TP63* gene in both Akagi and Parfenov studies highlight the importance of this gene in HNSCC tumorigenesis.

Interestingly, HPV integration could also affect infected cells epigenetically [55]. Hypomethylation and overexpression of BarH-like homeobox 2 (*BARX2*) and Iroquois homeobox protein 1 (*IRX4*) genes were shown in HPV-positive with integration status. These genes were shown to contribute to tumour formation [78, 79]. Conversely, hypermethylation and downregulation of Single-minded family bHLH transcription factor 2 (*SIM2*) and Cathepsin E (*CTSE*) were depicted in these cells. These genes are also shown to contribute to tumour formation [80, 81].

In a nutshell, the HPV virus integration process is a random yet cell-specific process leading to increased genetic alterations at the integration sites. Depending on the integration site, affected tumour suppressor genes could be downregulated/inactivated or affected oncogenes upregulated/activated. The integration process could also impact the epigenome and confer oncogenic properties. Integration-induced genetic and epigenetic changes are important determinants to what extent the pre-cancer cells require the viral oncoproteins E6 and E7 to drive the tumourigenesis process. Thus, the function of these oncogenes might not be required in all HNSCC patients.

Consistent with this postulation and, as mentioned earlier, the characterisation of oropharyngeal SCC tumours at single-cell resolution has unveiled the presence of a distinct subset of HPV-positive cells in which the expression of HPV is either lost or diminished. These cells are referred to as *HPVoff*, in contrast to the HPV-positive cells that continue to express E6 and E7 oncoproteins, referred to as *HPVon*. The similar genomic copy number of HPV genes between *HPVon* and *HPVoff* cells indicates that epigenetic regulation controls HPV gene expression in *on* and *off* subclones. This was concluded based on in vitro results following the inhibition of enhancer of zeste 2 polycomb repressive complex 2 subunit (EZH2) and DNA methyltransferase (DNMT) that result in the reduction of HPV gene expression [62]. While the findings were described in cancer cell lines rather than a progressive HNSCC model, the possibility of losing the HPV genomic content during cancer progression is not ruled out.

Intriguingly, functional enrichment analysis revealed a significant association of G1/S with *HPVon* cells compared to both *HPVoff* and HPV-negative cells. Conversely, *HPVoff* cells exhibited an enrichment of the epithelial senescence-associated (EpiSen) meta-programme compared to *HPVon* cells [62]. Consistent outcomes were concluded by analysing the bulk TCGA and HPV-positive cells (93VU147T and SCC47), supporting the concept that *HPVon* cells have higher proliferation and less senescence compared to *HPVoff* and HPV-negative cells. Importantly, *HPVoff* tumours also tend to have reduced recurrence-free survival. Further in vitro analysis of the *HPVon* and *HPVoff* subclones of 93VU147T and SCC47 revealed higher sensitivity of *HPVon* cells to therapeutic agents such as Cisplatin and radiation. *HPVoff* cells, however, showed treatment resistance and tend to migrate more significantly [62].

These findings suggest that *HPVon* may drive tumourigenesis by destabilising the genome and inhibiting key tumour suppressors such as p53 and Rb1. Consequently, a subset of HPV-positive malignant cells undergoes a loss of active transcription state, resulting in the suppression of hyperproliferative characteristics. This phenomenon ultimately confers resistance to conventional treatments for HPV-positive HNSCC, increases the risk of metastasis and worsens the prognosis for patients.

## Prognosis of HPV-positive HNSCC patients

Survival rates for HNSCC patients have seen modest improvement in the last 30 years. An analysis spanning numerous HNSCC subgroups revealed better survival rates across different age groups, except for patients over the age of 75, while survival rates have improved for most anatomical sites. The overall increase in survival can be partially attributed to the emergence of HPV-associated HNSCC, a subgroup that benefits from a better prognosis [20, 82].

With the HPV-positive HNSCC, a subset exhibits extremely unfavourable treatment outcomes similar to HPV-negative patients. Only a limited number of studies have provided insights into comprehending the molecular basis of this phenomenon. The presence of lymphocyte infiltration in HPV-positive tumours has demonstrated superior outcomes, while the absence of local infiltration by these cytotoxic cells mirrors the unfavourable survival observed in HPV-negative cases [37].

By single-cell analysis of immune and non-immune cell interactions in head and neck tumours, Kurten et al. showed no clear association between HPV status and inflammation, where among 6 HPV-positive samples, one was in the low inflammation, two were in the medium, and three were in the high group, confirming the heterogeneity of HPV-positive tumours and their microenvironment [83]. Nevertheless, it remains uncertain whether these patients have an insufficient adaptive immune response or, more likely, alterations within HNSCC with HPV integration (such as *TRAF3* and *PIK3CA* mutations) facilitate the evasion of tumour cells from the immune system. This is speculated to impart resistance against therapy and decreased survival rates for these patients.

**Fig. 4:**
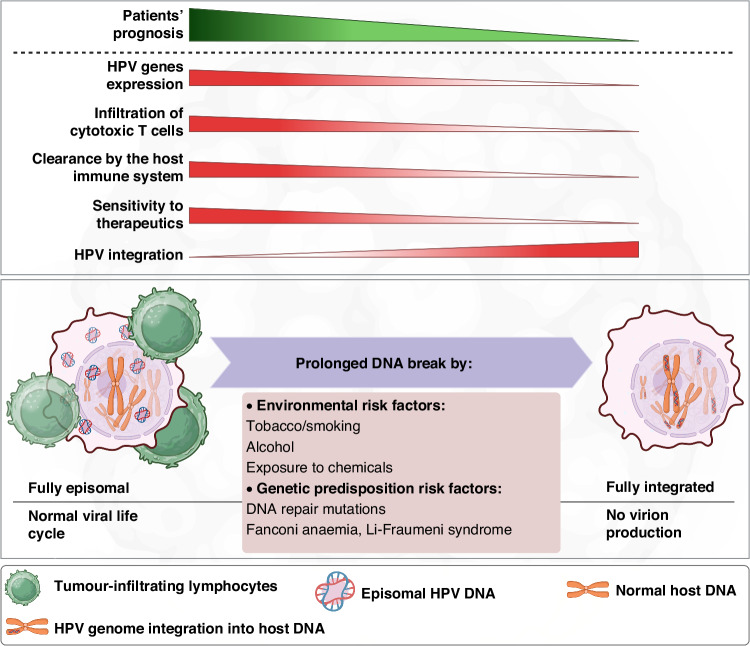
The proposed model describes the poor prognosis of HPV-positive HNSCC. HNSCC patients with high HPV genome integration show reduced HPV gene expression and lowered infiltration of T cells that result in unfavourable clinical outcomes similar to HPV-positive HNSCC. HPV human papillomavirus, HNSCC head and neck cancer squamous cell carcinoma.

The impact of HPV integration on reduced survival rates could be bolstered by reports indicating the prevalence of HPV genome integration within the genomes of high-grade HNSCCs [55, 56] and cervical cancers [57–60]. More importantly, elevated integration levels of HPV are reported to correlate with poorer survival of HNSCC patients [61]. These findings suggest that a higher frequency of integration, along with its downstream effects on the transcriptome and proteome of the host cell, could potentially alter the tumour microenvironment and promote immune evasion (Fig. 4).

Furthermore, there appears to be a correlation between HPV integration and the *HPVon*/*HPVoff* status presented by Puram et al. [62]. In fact, the link established between *HPVoff* status and reduced survival outcomes could potentially be attributed to increased integration events. Consistently, Zhang et al. demonstrated that an HPV subgroup with elevated levels of keratinocyte differentiation and oxidation-reduction process (HPV-KRT) has higher levels of viral integration and lower expression of viral genes E2/E4/E5, compared to the other HPV-positive samples with strong immune response and mesenchymal differentiation (HPV-IMU) [84]. This highlights the link between the integration status, expression states of the viral genes, and immune cell infiltration. To test this proposed hypothesis shown in Fig. 4, it would be pivotal to conduct an analysis on a large cohort of HPV-positive HNSCC samples with varying prognoses. The investigation would involve tracking the HPV genomics and transcriptomics content to determine the extent of integration in association with clinical outcomes. This can be complemented by tracing the TILs in samples with good or bad prognoses.

## Signalling pathways associated with HPV integration

Over the last few decades, there has been a notable surge in the incidence of HPV-positive HNSCCs, particularly oropharyngeal cancers. Besides, the prevalence of HPV-positive HNSCC is higher among younger individuals, imposing a greater economic, medical, and psychological burden on societies. Given that the integration of the HPV genome is recognised as a primary event in HPV-positive tumour development, it is crucial to identify and target the molecular pathways that control the integration process. Moreover, such an approach can enhance the survival of HPV-positive HNSCC patients by preventing additional integrations and maintaining the tumour cells in an episomal state, likely resembling an *HPNon* status. This strategy would help preserve the sensitivity of HPV-positive HNSCC to standard therapeutic interventions.

**Fig. 5:**
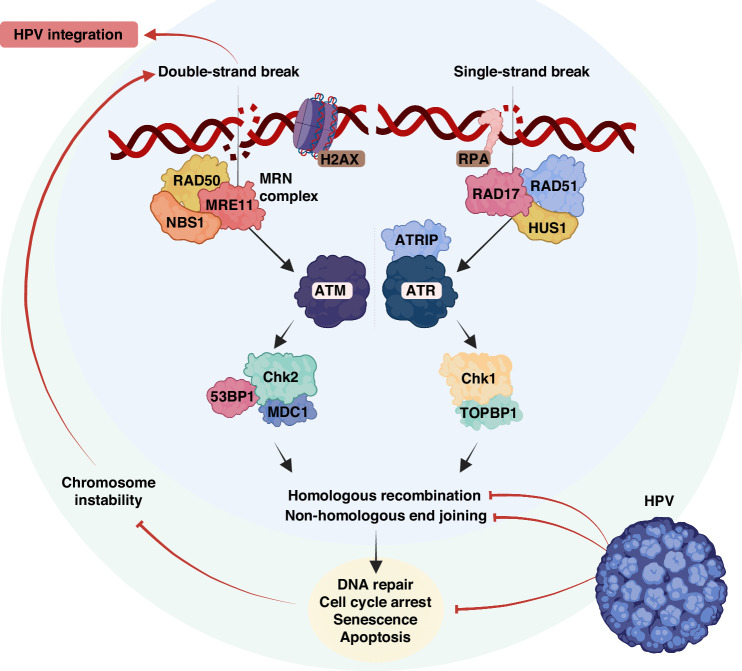
HPV perturbs DNA damage response signalling pathway. HPV infection could manipulate the normal DNA repair systems in the cells and thus enhance the level of chromosomal instability and DNA breakage. This phenomenon ultimately increases the rate of HPV integration into the host genome. HPV human papillomavirus, RAD DNA repair protein, MRE11 meiotic recombination 11 homologue A, NBS1 Nijmegen breakage syndrome 1, ATM ataxia telangiectasia mutated, Chk checkpoint kinase, 53BP1 p53-binding protein 1, MDC1 mediator of DNA damage checkpoint protein 1, RPA replication protein A, HUS haemolytic-uraemic syndrome, ATRIP ATR-interacting protein, ATR ataxia telangiectasia and Rad3-related protein, TOPBP1 DNA topoisomerase 2-binding protein 1.

The HPV genome integration into the host’s genome is still poorly understood. The current knowledge strongly suggests that DNA damage contributes to HPV integration. Eukaryotic cells sense the DNA damage and repair it mainly by Ataxia telangiectasia mutated (ATM), Ataxia telangiectasia and Rad3-related (ATR), and DNA protein kinases (Fig. 5). While ATM is activated in response to double-stranded breaks (DSBs), ATR responds to single-stranded lesions. DSBs can occur in chromosomal DNA due to replication fork collapse, programmed rearrangements, physical stress, or exposure to damaging agents like reactive oxygen and nitrogen species (ROS and RNS). These lesions pose a threat to cell viability and can lead to chromosomal translocations and genomic instability. DSBs can be repaired through two main pathways being the non-homologous end-joining (NHEJ) or homologous recombination (HR), depending on the availability of repair templates and the cell cycle stage [85, 86]. HR relies on the functions of the Rad51 family of proteins, while NHEJ is initiated by the association of Ku70/80 proteins with DNA ends and the recruitment of the DNA-dependent protein kinases [87]. Auto-phosphorylation of these kinases allows their release from DNA, enabling the processing of DNA ends for repair by DNA ligase IV and X-ray repair cross-complementing group (XRCC4).

The Mre11-Rad50-Nbs (MRN) complex is considered a sensor for DSBs [88]. It is crucial for effective activation of ATM, which undergoes auto-phosphorylation and then signals to numerous downstream targets involved in checkpoints and repair mechanisms [89]. The DNA damage cascade phosphorylates various proteins, including mediators (53BP1 and Mdc1) and effectors (Chk1 and Chk2) of checkpoint responses. One of the initial proteins to be phosphorylated upon DNA damage is the histone variant H2AX, which acts as a signal for recruiting DNA damage proteins to DSBs [90]. ATR is specifically activated during the S phase to regulate the firing of origins and repair damaged replication forks [91]. ATR is recruited to sites of single-stranded DNA (ssDNA) damage through its association with ATR-interacting protein (ATRIP), which recognises the RPA complex coating ssDNA. The kinase activity of ATR-ATRIP is stimulated by TopBP1 [92]. Upon recruitment, ATR phosphorylates substrates, including Chk1 for checkpoint activation, and RPA32, Smc1, and Rad9 to facilitate repair processes [93]. Viral interactions with components of these networks are likely to unveil regulatory processes and determine the pathways exploited or inactivated. For instance, silencing a key regulator of NHEJ, Ku70, resulted in the generation of double-strand DNA breaks in episomal W12 cells and was shown to induce new HPV16 viral integration events [94]. Consistently, Someya et al. reported a significantly lower activity of DNA-dependent protein kinases Ku70 and Ku86 in cervical cancer patients compared to normal volunteers [95]. These findings suggest that enhancing DSBs is a primary mechanism HPV employs to integrate into the host cell genome.

The most deregulated pathway in HNSCC, i.e., PI3K-AKT-mTOR, could be another bona fide target to prevent viral integration. In HNSCC tumours, genetic alterations in components of this pathway are frequently observed [20, 48, 96]. *PIK3CA*, the catalytic domain of PI3K kinase undergoes mutations and gene amplifications at high frequency in HNP-positive HNSCC. Furthermore, loss of function in PTEN, which negatively regulates the PI3K-AKT signalling, is identified in numerous HNSCC tumours, resulting from both genetic and epigenetic changes [20, 97]. A systematic review of 95 HNSCC patients presented that 39% of cases with *PIK3CA* mutations are associated with nearly 5 times increase in the risk of recurrence or death in HPV-positive patients [98]. Furthermore, it is reported that the HPV16 E7 protein can promote the phosphorylation of Akt, even in the absence of PTEN downregulation. E7 is known to physically bind and sequester the PP2A protein, which in turn maintains the activation of the PI3K/Akt signalling pathway [99]. On the other hand, E6 protein can activate mTOR complex 1 leading to oncogenic activation of the PI3K-AKT-mTOR pathway [100]. Although there are no reports elaborating on the exact mechanism by which the PI3K-AKT-mTOR pathway causes HPV integration, it appears that constitutive activation of this oncogenic pathway maintains cellular transformation and increases the rate of DNA breakages over multiple uncontrolled cell divisions.

## Future directions and perspectives

HPV-positive HNSCC cases are expected to continue to rise. HPV-positive patients generally exhibit improved survival rates compared to HPV-negative patients, but a subset of HPV-positive HNSCC patients with high HPV integration levels experience poorer prognostic outcomes. The HPV integration is a dynamic process occurring years before tumour formation which leads to genetic alterations, potentially activating oncogenes and inactivating tumour suppressors. The integration-induced genomic instability and altered host gene expression play a key role in the initiation and sustained promotion of cancer growth.

Furthermore, dynamic HPV integration plays a pivotal role in shaping the diverse expression profiles of HPV genes within the host cell. Typically, HPV integration events have been associated with the disruption of the viral E2 gene and the subsequent overexpression of oncogenic E6 and E7 genes at the initial stage of HPV-positive tumour development. As the tumour evolves over time, increasing genetic changes are driven by heightened HPV integration levels which further promote viral gene expression. This phenomenon can result in the loss of sensitivity to standard therapies, allowing the tumour to persist and recur.

Despite HPV-positive HNSCC being characterised as immune-hot, the process of HPV integration can significantly impact the tumour microenvironment. While oncogenic viral genes E6 and E7 are highly expressed, viral genes responsible for viral replication and transcription, such as E1^4 and E2, are either absent or expressed at low levels in HNSCC with HPV integration. More importantly, viral antigen E2 is known to strongly trigger the activation of antiviral antibody-secreting cells and T cells, responsible for viral clearance. This suggests that loss of E2 during HPV integration contributes to the immune evasion of HPV-positive tumour cells.

It is now clear that HPV integration is responsible for *de-novo* genetic alterations, diverse HPV gene expression patterns, and a tumour-permissive microenvironment, ultimately contributing to the heterogeneity of HPV-positive HNSCC. Given the significant role of HPV integration in the development and progression of HNSCC, targeting this process holds significant therapeutic implications. Inhibiting HPV integration could maintain the episomal state of the viral genome, reducing the oncogenic effects of integrated E6 and E7 oncogenes, and preserving the sensitivity of tumour cells to standard therapies.

One potential therapeutic strategy to inhibit HPV integration into the host genome involves targeting the DNA repair mechanism. Genes essential for the repair of DNA DSB are frequently lost or highly mutated in HNSCC with integrated HPV, proposing vulnerabilities of these tumours to therapies that exploit DNA repair defects, such as Poly(ADP-ribose) polymerase (PARP) inhibitors. Indeed, clinical trials are currently investigating the use of PARP inhibitor Olaparib as radiosensitisers to enhance local control in patients with advanced HPV-positive HNSCC, highlighting the significance of understanding the intricate interactions between HPV integration and DNA repair pathways towards developing novel treatment strategies for HPV-positive cancers.

Overall, fundamental research and preclinical studies aiming to better understand the molecular mechanisms of HPV integration and its impact on HNSCC progression are urgently needed. Ultimately, therapeutic strategies that prevent HPV integration may offer promising opportunities for heterogeneous HPV-positive HNSCC, consequently improving the management and outcomes for HPV-positive HNSCC patients.

## Data Availability

Not applicable.
